# Editorial: Striking the Right Balance for Eligibility Criteria for Clinical Trials in Inflammatory Bowel Disease

**DOI:** 10.1002/ueg2.12749

**Published:** 2025-01-06

**Authors:** Virginia Solitano, Vipul Jairath, Silvio Danese

**Affiliations:** ^1^ Department of Medicine Division of Gastroenterology Western University London Canada; ^2^ Departments of Medicine, Epidemiology and Biostatistics Western University London Canada; ^3^ Division of Gastroenterology and Gastrointestinal Endoscopy IRCCS Ospedale San Raffaele University Vita‐Salute San Raffaele Milan Italy

Eligibility criteria for regulatory clinical trials are important for both internal and external validity and ultimately will inform drug labeling and access. However, the restrictive nature of eligibility criteria has long been criticized for limiting the generalizability of trial findings to real‐world practice [1]. There is a common misunderstanding among many clinicians that clinical trials designed for regulatory purposes should reflect the patients they treat in their own practice, implying they should have external validity and generalizability. However, trials designed for drug approval are intended to be explanatory in nature, conducted in homogeneous populations defined by specific eligibility criteria. To determine if treatments are effective in various patient subgroups, either phase 4 trials or carefully designed real‐world studies are necessary. Nonetheless as medicine appropriately so evolves toward a more inclusive, patient‐centered paradigm, it is essential to re‐evaluate how eligibility criteria can be broadened without compromising the scientific rigor and ethical standards of clinical trials [2, 3].

We read with interest the paper recently published in the United European Gastroenterology Journal by Outtier and colleagues, analyzing eligibility criteria in phase 3 placebo‐controlled randomized controlled trials (RCTs) for moderate‐to‐severe inflammatory bowel disease (IBD), over 22 years (2000–2022). Quantitative analysis of almost 60 RCTs revealed a median of 44 eligibility criteria per trial, which remained consistent over time. Qualitatively, the analysis highlighted frequent exclusion of specific patient populations, such as older adults, therapy‐refractory patients, those with comorbidities, prior malignancies, unclassified IBD, ulcerative proctitis, stricturing and fistulizing CD, or ostomies. These findings highlighted the restrictive and complex nature of eligibility criteria in IBD clinical trials, which may limit the applicability of trial results.

Restrictive eligibility criteria are implemented to avoid known or unknown confounding variables that could affect the efficacy or safety of an investigational product. This approach is particularly crucial in earlier‐phase trials, where minimizing risks to participants and isolating treatment effects are paramount. For regulators, these measures ensure that submitted data reflects a robust and scientifically sound evaluation of the intervention under controlled conditions.

Restrictive criteria can protect safety by excluding high‐risk populations, reducing adverse events and trial failure. Standardizing parameters, such as wash‐out periods and disease activity levels, ensure consistent treatment responses and endpoint evaluations.

Despite these advantages, restrictive eligibility criteria can lead to a subset of patients different from the diverse demographics encountered in routine practice.

As clearly shown by the authors older adults, difficult‐to‐treat patients, individuals with comorbidities or prior malignancies, pregnant women, those of diverse ethnicity, and those living with ostomies are frequently excluded, despite being the very groups who might benefit most from new treatments [4, 5, 6].

Such exclusions create a gap between trial efficacy and real‐world effectiveness. When therapies transition from the controlled environment of RCTs to the complexities of clinical practice, unanticipated challenges often arise [7]. This misalignment can lead to difficulties in interpreting trial data, limited applicability to broader patient populations, and even regulatory hesitation when granting approvals for expanded indications.

There is an increasing acknowledgment that clinical trials need to adapt in order to better represent the populations they aim to benefit from. Regulatory initiatives highlight the importance of including underrepresented groups in research to ensure more inclusive and applicable results [8, 9]. A recent initiative by the International Organization for the Study of Inflammatory Bowel Diseases (IOIBD) developed 26 evidence‐based recommendations for broadening IBD clinical trial eligibility. Key recommendations include including patients with ulcerative proctitis, unclassified colitis, and stricturing or perianal CD, as well as patients with HIV or prior malignancies with appropriate evaluations [10]. It is important that the appropriate endpoints for measuring response to therapy in these niche subtypes have been developed and validated.

A balanced solution lies in adopting a tiered approach to trial design. Early‐phase trials might retain stricter eligibility criteria to ensure safety and establish proof of concept, while later‐phase studies could gradually broaden inclusion to capture diverse patient populations, capping subsets of disease such as proctitis, stricturing, and fistulizing CD. Additionally, regulators should encourage hybrid approaches that integrate rigorously designed real‐world evidence studies, post‐approval studies, and registries alongside traditional RCTs.

In conclusion, the debate over eligibility criteria is not about choosing between restriction and inclusion but finding the right balance (Figure 1). Restrictive eligibility criteria have their place in safeguarding trial integrity and participant safety, but they must not become barriers to generalizability. By embracing broader criteria, adaptive designs, and regulatory flexibility, clinical trials can better align with the needs of real‐world practice, ultimately delivering therapies that are not only effective but also equitable and accessible.

**FIGURE 1:**
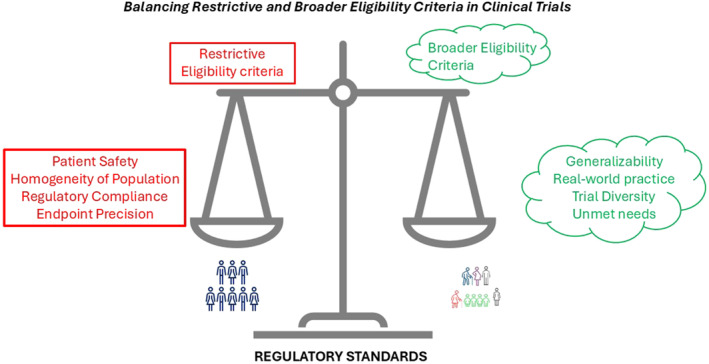
Balancing eligibility criteria in IBD trials.

## Conflicts of Interest

VS has received speaker fees from Pfizer. VJ has received consultancy/advisory board fees from AbbVie, Alimentiv Inc., Arena pharmaceuticals, Asahi Kasei Pharma, Asieris, Astra Zeneca, Bristol Myers Squibb, Celltrion, Eli Lilly, Ferring, Flagship Pioneering, Fresenius Kabi, Galapagos, GlaxoSmithKline, Genentech, Gilead, Janssen, Merck, Mylan, Pandion, Pendopharm, Pfizer, Protagonist, Reistone Biopharma, Roche, Sandoz, Second Genome, Takeda, Teva, Topivert, Ventyx, and Vividion; and speaker’s fees from, Abbvie, Ferring, Galapagos, Janssen Pfizer Shire, Takeda, and Fresenius Kabi. SD has received consultancy fees from AbbVie, Alimentiv, Allergan, Amgen, AstraZeneca, Athos Therapeutics, Biogen, Boehringer Ingelheim, Bristol Myers Squibb, Celgene, Celltrion, Dr Falk Pharma, Eli Lilly, Enthera, Ferring Pharmaceuticals Inc, Gilead, Hospira, Inotrem, Janssen, Johnson & Johnson, MSD, Mundipharma, Mylan, Pfizer, Roche, Sandoz, Sublimity Therapeutics, Takeda, TiGenixa, UCB Inc, and Vifor; and lecture fees from AbbVie, Amgen, Ferring Pharmaceuticals Inc, Gilead, Janssen, Mylan, Pfizer, and Takeda.

## Data Availability

Data sharing is not applicable to this article as no new data were created or analyzed in this study.
